# Melanuria without Melanotic Tumor

**Published:** 1913-12

**Authors:** 


MELANURIA WITHOUT MELANOTIC TUMOR. Zoeppritz, *Münch. Med. Woch,* No. 23, 1911, reports the case of a man of 69, suffering with multiple abscesses, later developing ileo-psoas abscess with perforation into the peritoneum and death. The urine, yellowish-brown as passed, gradually became black and opaque on standing and gave the characteristic tests for melanin. Necropsy revealed a small annular carcinoma of the sigmoid, with metastases to the meso-sigmoid and peritoneum but no pigment deposits. Phenol, salol and coal-tar products were excluded. There was no nephritis as in the case of Gaezda, who considered the increased renal permeability the cause of the melanogenuria. Helman found that 7.3 per cent. of melanotic tumors showed melanuria. Melanuria without tumor is exceedingly rare, and the possibility of an undiscovered small melanotic tumor must be borne in mind.

